# Intelligence

**Published:** 1830-05

**Authors:** 


					INTELLIGENCE.
MR. WALLERS OBSTETRICAL PRIZE.
The public examination prior to the presentation of this prize took place i?
the Theatre of the Aldersgate street School, on Saturday, April 24th. I*
lasted for upwards of four hours; at the expiration of which period, Mr. W*
J. E. Wilson, of Greenhithe, Kent, was declared to be the successful candi-
date. The examination was conducted by the lecturer himself, and Messrs-
Watson, Doubleday, and Rushforth, (three medical practitioners,)
acted as umpires on the occasion. The answers to the questions proposed*
which embraced the principal points connected with the art and science
midwifery, were highly creditable to the pupils, and must have been equally
gratifying to the teacher.
469
HUNTERIAN MEDAL.
We are requested to say, that the competition for the prize offered by the
Hunterian Society for the best essay on Tubercular Affections, is not limited
'o members.
LIBRARIAN TO THE KING.
il'e Jate Dr. Gooch was the first member of our profession who held the
ce of librarian to the King. The vacancy occasioned by his death has
J,lst been filled up by the appointment of Dr. Macmiciiael; and we have
pleasure in mentioning the nomination of that gentleman, both because an
nourable mark of distinction is thus retained in the profession, and because
1 ,s bestowed on one whose accomplishments and character entitle him to the
respect and esteem of his brethren.?Med. Gaz.
r* Robert Lee, physician to the Brownlovv street Hospital, has been
e ected a Fellow of the Royal Society.
ajmuel Brougiiton, Esq. surgeon to the 2d Regiment of Life Guards,
f.n *'le St. George's and St. James's Dispensary, has been elected a Fellow of
1(1 Royal Society.
472 INTELLIGENCE.
A Manual of Descriptive Anatomy of the Human Body ; illustrated by Litho-
graphic Plates. By J. Cloquet. Translated from the French by Thomas
King, late House Surgeon to the Hdtel Dieu, and Lecturer on Anatomy at the
Aldersgate-street Medical School.
The celebrity of Cloquet as an anatomist is well known, and his Manual
is considered to be one of the most valuable and instructive works of the pre-
sent day. In Mr. King he has a very able translator, from his intimate
knowledge of the language, and his perfect acquaintance with the science of
anatomy. In its original language, this excellent work could have been but
of little use to the majority of English students; who, however capable they
may generally be of reading the French language, would fail in perfectly
comprehending the relative technical terms. Mr. King's translation possesses
a double advantage: the original text and the English translation are given
in separate columns, and thus the student, while he is perfecting himself in
anatomy, will at the same time make himself master of the French anatomical
terms, which he could scarcely do by any other mode of instruction.
The work is to be published in a series of parts; and, from the excellence
and accuracy of the illustrative plates, and the descriptive text, of the first
part, which we have received as a specimen, we warmly recommend it to our
junior brethren, and to those who are desirous of renewing their lost anato-
mical knowledge.
Lectures on Practical and Medical Surgery. By Thomas Alcock.
In this work the student will find much wise and sensible counsel respecting
his surgical education and professional conduct. The remarks of the author
on the investigation of disease are very judicious; and the comments on the
occasional ill consequences of venesection, and on bloodletting from the veins
of the lower extremity and from the external jugular vein, deserve particular
attention. A very interesting outline is also contained in the work of the
general anatomy of the mucous membranes. The regulations proposed by
Mr. Alcock for the guidance of his pupils in the performance and explanation
of a series of surgical operations, are well calculated to further the grand
object of professional improvement.
Dr. Uwins will publish, in the course of a few days, a pamphlet on " Ner-
vous and Mental Disorder," with especial reference to recent investigations
on the subject of Insanity.
Dr. Ure has in the press a new edition, nearly rewritten, of his Dictionary
of Chemistry.
METEOROLOGICAL JOURNAL,
By Messrs. Harris and Co. Mathematical Instrument Makers, 50, High Hoi born.
20
21
22
23
24
25
26
27
28
29
30
31
April
2
3
4
5
6
7
8
9
10
11
12
13
14
15
16
17
18
19
.55
o
Barometer,
30.00
.07
.04
29.92
.97
30.21
.40
.44
.24
.09
29.90
.66
.57
.34
.09
.86
30.05
9.66
.74
.48
.37
.30
.47
.32
.64
.90
.66
.50
.54
.52
.49
30.07
.05
29.78
.92
30.10
.28
.43
.36
.11
29.95
.86
.60
.60
.23
.70
.97
.89
.69
.69
.39
.32
.52
.36
.46
.80
.84
.57
.60
.57
.50
.61
DeLuc':
Hygrom
Winds.
VV
s\v
VV
WNW
VV
W
VV
w
SE
S
SSVV
ESE
NE
E
N
NVV
NE
VV
vvsw
s
SSVV
VV
svv
VVNVV
VV
VVNVV
sw
VV
svv
vvsw
w
VVNVV
sw
s w
vvsw
NVV
w
VV
VV
SE
SSVV
ESE
NE
E
NE
W
NVV
VVSW
VVNVV
SW
s
SSVV
svv
svv
w
VVNVV
svv
svv
VVS VV
VV
VVSW
w
Atmospheric Variations.
Fine
Cloudy
Fine
Foggy
Fine
Foggy
Fine
Foggy
Rain
Sleet
Sleet
Fine
Foggy
Fine
Cloudy
Fine
Cloudy
Fine
Cloudy
Cloudy
Foggy
Sleet
Cloudy
Cloudy
Fine
Cloudy
2 p.m. 10 p.m.
Fine
Cloudy
Cloudy
Fine
Rain
Rain
Cloudy
Fine
Cloudy
Rain
Cloudy
Fine
Cloudy
Clou iy
Show'ry
Fine
Cloudy
The Rain gauge having been damaged by the late frost, the quantity for March cannot be
given.

				

